# Linear growth is independently associated with health-related quality of life during healthcare transition in youth with special health care needs

**DOI:** 10.3389/fnut.2026.1905544

**Published:** 2026-08-03

**Authors:** Patrícia Zamberlan, Clara Doria, Ana Clara S. R. Praxedes, Ana Paula Scoleze Ferrer, Darusa C. Souza, Claudia B. Fonseca, Flávia C. Yarshell, Pedro Henrique P. Sampaio, Michele Luglio, Bruno Gualano, Rafael Almeida Azevedo, Katia Kozu, Nadia Emi Aikawa, Lucia Maria Arruda Campos, Andreia Watanabe, Marlene P. Garanito, Luiz Vicente R. F. Silva Filho, Ricardo Katsuya Toma, Caroline G. B. Passone, Louise Cominato, Antônio Carlos Pastorino, Clovis Artur Silva, Artur Delgado

**Affiliations:** Department of Pediatrics, Hospital das Clínicas, São Paulo University, São Paulo, Brazil

**Keywords:** adolescents, chronic disease, nutritional status, quality of life, elf-management, transition to adult care

## Abstract

**Introduction:**

Youth with special health care needs (YSHCN) are particularly vulnerable during the transition from pediatric to adult healthcare. Although nutritional status is a potentially modifiable determinant of health, its relationship with transition readiness and health-related quality of life (HRQoL) during this period remains poorly understood.

**Objective:**

To investigate the association of nutritional status and socioeconomic factors with transition readiness and HRQoL in YSHCN undergoing healthcare transition.

**Methods:**

This cross-sectional study included 218 consecutive YSHCN followed at a tertiary university hospital in Brazil. Transition readiness was assessed using the Transition Readiness Assessment Questionnaire (TRAQ), and HRQoL was evaluated using the Pediatric Quality of Life Inventory 4.0 (PedsQL 4.0). Nutritional status was assessed using body mass index-for-age (zBMI/A) and height-for-age (zH/A) z-scores according to World Health Organization standards. Multivariable linear regression models were used to identify factors independently associated with TRAQ and PedsQL scores. Logistic regression analyses were performed to identify predictors of low transition readiness (TRAQ <3.5).

**Results:**

Nutritional status assessed by BMI-for-age (zBMI/A) was not associated with transition readiness or HRQoL. In multivariable analyses, female sex (*β* = 0.18, *p* = 0.002) and older age (*β* = 0.12, *p* = 0.001) were independently associated with higher TRAQ scores. Higher height-for-age (zH/A) (*β* = 1.86, *p* = 0.031) and higher family income (*β* = 2.87, *p* = 0.025) were independently associated with better HRQoL, whereas female sex was associated with lower HRQoL (*β* = −7.25, *p* = 0.001). In logistic regression analyses, younger age (OR = 0.82, 95% CI 0.70–0.96, *p* = 0.023) and male sex (OR = 0.48, 95% CI 0.25–0.92, *p* = 0.023) were significant predictors of low transition readiness, while nutritional risk was not associated with the outcome. The logistic model demonstrated modest discrimination (AUC = 0.689).

**Conclusion:**

In YSHCN undergoing healthcare transition, BMI-for-age was not associated with transition readiness or HRQoL. In contrast, linear growth and family income were independently associated with better HRQoL, highlighting the importance of developmental and socioeconomic factors in patient-reported outcomes. These findings reinforce the multifactorial nature of transition readiness and HRQoL and support the incorporation of nutritional, developmental, and psychosocial assessment into multidisciplinary healthcare transition programs.

## Introduction

Youth with special health care needs (YSHCN) represent a heterogeneous population of adolescents living with chronic physical, developmental, or behavioral conditions that require ongoing and complex health care. As survival into adulthood continues to improve, the transition from pediatric to adult-oriented health systems has emerged as a critical component of long-term care (1, 2).

Transition readiness refers to the ability of adolescents and young adults to manage key aspects of their health, including understanding their chronic condition, adherence to drug treatment and appointments, navigating healthcare systems, and engaging in self-advocacy. Inadequate transition readiness has been associated with poor treatment adherence, increased healthcare utilization, and worse clinical outcomes (3, 4). In addition, the transition period represents a phase of increased vulnerability, with risks of discontinuity of care and deterioration in health outcomes when not adequately supported (5).

Standardized instruments, such as the Transition Readiness Assessment Questionnaire (TRAQ), are widely used to assess self-management and self-advocacy skills during this process (3, 6). In parallel, health-related quality of life (HRQoL) has become a key patient-centered outcome, capturing the broader impact of chronic disease on physical, emotional, and social well-being (7, 8).

Despite advances in structured transition frameworks, including the Six Core Elements of Health Care Transition, important determinants of successful transition outcomes remain insufficiently explored (2, 9). Current evidence suggests that transition outcomes are influenced by a complex interplay of patient-, provider-, and system-level factors, including access to care, care coordination, and individual preparedness (10).

In particular, nutritional status, an essential and potentially modifiable component of health, has received limited attention during this transition process. Adolescents with chronic conditions are at increased risk of both undernutrition and excess weight due to disease-related metabolic demands, functional limitations, medication effects, and lifestyle factors, as sedentary behavior. These nutritional abnormalities may directly impact physical capacity, cognitive function, treatment adherence, and psychosocial well-being. Moreover, nutritional status may reflect underlying disease complexity, often associated with polypharmacy, multispecialty care, and higher healthcare needs (11–15).

In addition, adolescents with chronic conditions are particularly vulnerable during the transition period and often require coordinated, multidisciplinary support that extends beyond medical care alone. Social determinants of health, including socioeconomic status, play a critical role in shaping transition outcomes, access to care, and long-term health trajectories. YSHCN frequently require integrated care approaches that combine medical, psychosocial, and social support throughout the transition process (16).

Although some transition frameworks and assessment tools incorporate nutritional counseling as part of healthy lifestyle promotion, current evidence does not establish whether baseline nutritional status is associated with transition readiness or HRQoL in YSHCN (7, 17).

Therefore, this study aimed to investigate whether nutritional status and socioeconomic factors are associated with transition readiness and health-related quality of life (HRQoL) in YSHCN undergoing healthcare transition.

## Methods

### Study design and participants

This study was approved by the institutional Ethics Committee (CAEE 84030924.4.0000.0068). Between March 2025 and April 2026, consecutive eligible youth with special health care needs attending routine outpatient visits at multiple pediatric specialty clinics within the institutional healthcare transition program of a tertiary university hospital were invited to participate, regardless of their underlying chronic condition. The cohort represented a broad spectrum of chronic and complex conditions managed across different pediatric specialties, reflecting the multidisciplinary nature of the institutional healthcare transition program.

A convenience sample was used, comprising all consecutive eligible participants attending the institutional healthcare transition program during the study period. No formal sample size calculation was performed because the study aimed to include the entire accessible population within the predefined recruitment period.

### Clinical and transition assessment tools

Participants were classified as YSHCN according to the National Survey of Children’s Health (NSCH) criteria (18). Transition readiness was assessed using the TRAQ, validated in Brazilian Portuguese and scored on a 1–5 Likert scale, with higher scores indicating greater readiness (3).

HRQoL was evaluated using the Pediatric Quality of Life Inventory 4.0 (PedsQL 4.0), with scores ranging from 0 to 100, where higher scores indicate better HRQoL (7).

### Nutritional assessment

Anthropometric measurements (weight and height) were obtained using standardized procedures. Body mass index (BMI) was calculated as weight (kg) divided by height squared (m^2^).

Nutritional status was assessed using age- and sex-specific z-scores based on the World Health Organization (WHO) reference standards (19, 20).

Nutritional status was analyzed using following complementary approaches:

- Continuous variables: BMI-for-age z-score (zBMI/A) and height-for-age z-score (zH/A);
- Categorical classification: undernutrition (zBMI/A < −2), eutrophic (zBMI/A between −2 and +1), and weight excess, including overweight and obesity (zBMI/A > +1);
- Stunting: defined as zH/A < −2;
- Nutritional risk: defined as the presence of at least one of the following conditions: undernutrition, excess weight, or stunting.

### Physical activity

Physical activity was assessed using the International Physical Activity Questionnaire (IPAQ) (21). Participants were classified as physically active or inactive based on the IPAQ classification, with those classified as “active” or “very active” considered physically active.

### Family income

Self-reported family income was categorized as ≤2, >2–4, and >4 Brazilian minimum wages. For regression analyses, family income was modeled as an ordinal variable, assuming a linear association across increasing income categories.

### Variables

The dependent variables were the total TRAQ and PedsQL scores, as well as the TRAQ domains (medication management, appointment keeping, and tracking health) (3) and the PedsQL domains (physical, emotional, social, and school functioning) (7).

To facilitate clinical interpretation, TRAQ scores were also dichotomized into low and high transition readiness, with low transition readiness defined as a TRAQ score <3.5.

### Covariates

Potential confounders included age, sex, family income, physical activity level, number of medications (used as a proxy for clinical complexity), and number of pediatric specialties involved in care. Family income was evaluated as a potential confounder in both the HRQoL and transition readiness regression models.

### Statistical analysis

#### Descriptive and bivariate analysis

Continuous variables were expressed as median (interquartile range [IQR]) and categorical variables as frequencies (%). Group comparisons were performed using the Mann–Whitney U test for continuous variables and Pearson’s chi-square or Fisher’s exact tests for categorical variables.

Participants with missing data in key study variables were excluded from the multivariable analyses. Complete-case analyses were performed, and no data imputation was undertaken.

#### Multivariable regression models

Multivariable regression models were constructed to evaluate the independent association between nutritional status and study outcomes.

For continuous variables, linear regression analyses were performed using TRAQ total score and PedsQL total score as dependent variables, with zBMI/A and zH/A included as primary independent variables. All multivariable models were adjusted for age, sex, family income, physical activity level, number of medications, and number of pediatric specialties. Results were reported as *β* coefficients with corresponding 95% confidence intervals.

Logistic regression models were also developed using low transition readiness as the dependent variable. Nutritional risk was included as the main exposure variable. Adjusted odds ratios (OR) and 95% confidence intervals (CI) were calculated.

For the logistic regression model, discrimination for identifying low transition readiness (TRAQ < 3.5) was evaluated using the area under the receiver operating characteristic curve (AUC).

#### Additional analyses

Exploratory analyses were conducted among participants with abnormal nutritional status to examine the distribution of socioeconomic characteristics across nutritional status categories.

#### Model diagnostics

Multicollinearity was assessed using variance inflation factors (VIF). Residual diagnostics, including inspection of residual plots and normal Q-Q plots, were performed to assess homoscedasticity and normality of residuals for the linear regression models. For the logistic regression model, calibration was assessed using the Hosmer–Lemeshow goodness-of-fit test, and discrimination was evaluated using the area under the receiver operating characteristic curve (AUC).

A two-tailed *p*-value < 0.05 was considered statistically significant. Analyses were performed using IBM SPSS Statistics, version 27.0 (IBM Corp., Armonk, NY, USA).

### Artificial intelligence assistance

Artificial intelligence (ChatGPT; OpenAI) was used to assist with English language editing and to improve the presentation and reporting of the statistical analyses. All AI-generated suggestions were critically reviewed, verified, and revised by the authors, who take full responsibility for the final content of the manuscript.

## Results

### Baseline characteristics and nutritional status

Of the 221 eligible participants, three were excluded because of missing data in key study variables (PedsQL or anthropometric measurements), resulting in a final analytical sample of 218 participants (105 males and 113 females). Baseline demographic and nutritional characteristics, as well as the pediatric specialties involved, are presented in Table 1.

**Table 1:** Baseline characteristics of YSHCN according to sex.

Variables	Male (*n* = 105)	Female (*n* = 113)	*p*-value
Demographic			
Age (years)	16.3 (15.6–17.2)	16.4 (15.3–17.5)	0.746
Nutritional status			
Weight (kg)	58.0 (50.3–68.8)	57.2 (49.0–65.0)	0.228
Height (cm)	167.0 (160.5–173.0)	158.0 (154.0–162.0)	<0.001
BMI/A (kg/m^2^)	20.5 (18.8–24.1)	22.5 (19.9–26.1)	0.014
zBMI/A	−0.0 (−0.8–1.0)	0.5 (−0.3–1.4)	0.017
zH/A	−0.8 (−1.6 – −0.1)	−0.7 (−1.2–0.0)	0.135
zBMI/A classification			0.125
Undernutrition	9 (8.4%)	4 (3.5%)	
Eutrophic	70 (65.4%)	69 (60.5%)	
Weight excess	28 (26.2%)	41 (36.0%)	
zH/A classification			0.333
Stunting	18 (16.8%)	13 (11.4%)	
Normal height	89 (83.2%)	101 (88.6%)	
Nutritional risk			0.787
Yes	46 (43.0%)	52 (45.6%)	
No	61 (57.0%)	62 (54.4%)	
Pediatric specialties			
Cardiology	0 (0.0%)	1 (0.9%)	1.000
Endocrinology	8 (7.6%)	12 (10.6%)	0.489
Gastroenterology/Nutrology	13 (12.4%)	9 (8.0%)	0.369
Genetics	1 (1.0%)	0 (0.0%)	0.482
Hematology	12 (11.4%)	12 (10.6%)	1.000
Hepatology and liver transplantation	11 (10.5%)	20 (17.7%)	0.174
Immunology and allergy	5 (4.8%)	8 (7.1%)	0.573
Nephrology and renal transplantation	22 (21.0%)	17 (15.0%)	0.291
Neurology	5 (4.8%)	6 (5.3%)	1.000
Pediatric surgery	1 (1.0%)	1 (0.9%)	1.000
Pneumology	15 (14.3%)	6 (5.3%)	0.037
Psychiatry	3 (2.9%)	2 (1.8%)	0.674
Rheumatology	18 (17.1%)	36 (31.9%)	0.013

BMI, body mass index; zBMI/A, BMI-for-age z-score; zH/A, height-for-age z-score.

Stunting was observed in a minority of participants and approximately 45% of the sample met criteria for nutritional risk, with comparable proportions between males and females.

Females demonstrated significantly higher transition readiness (TRAQ) scores and lower HRQoL scores than males (all *p* < 0.05).

### Multivariable analysis

In multivariable linear regression analyses adjusted for age, sex, family income, physical activity level, number of medications, and number of pediatric specialties (Table 2). Neither zBMI/A (*β* = 0.02, *p* = 0.319) nor zH/A (*β* = 0.01, *p* = 0.688) was independently associated with TRAQ scores. Female sex (*p* = 0.002) and older age (*p* = 0.001) were independently associated with higher TRAQ scores. Physical activity was not significantly associated with either transition readiness or HRQoL.

**Table 2:** Multivariable linear regression for transition readiness measured using TRAQ.

Variable	*β*	95% CI	*p*-value
zBMI/A	0.02	−0.02–0.06	0.319
zH/A	0.01	−0.03–0.05	0.688
Female sex	0.18	0.07–0.29	**0.002**
Age	0.12	0.05–0.19	**0.001**
Physical activity (active)	0.09	−0.01–0.19	0.081
Number of medications (polypharmacy)	−0.01	−0.03–0.01	0.287
Number of specialties	−0.02	−0.06–0.02	0.314

BMI, body mass index; zBMI/A, BMI-for-age z-score; zH/A, height-for-age z-score. Bold values indicate statistically significant results (*p* < 0.05).

In the multivariable linear regression model adjusted for age, sex, family income, physical activity level, number of medications, and number of pediatric specialties (Table 3), zBMI/A was not independently associated with HRQoL, whereas higher zH/A was independently associated with better HRQoL (*β* = 1.86, *p* = 0.031). Female sex remained strongly associated with lower HRQoL (*β* = −7.25, *p* = 0.001). In addition, higher family income was independently associated with better HRQoL (*β* = 2.87, *p* = 0.025).

**Table 3:** Multivariable linear regression for health-related quality of life measured using PedsQL 4.0.

Variable	*β*	95% CI	*p*-value
zBMI/A	−1.07	−2.59–0.44	0.164
zH/A	1.86	0.17–3.55	**0.031**
Female sex	−7.25	−11.57 to −2.93	**0.001**
Age	−0.04	−0.19–0.11	0.602
Family income	2.87	0.36–5.39	**0.025**
Physical activity (active)	3.80	−0.40–8.00	0.079
Number of medications	−0.60	−1.40–0.20	0.150
Number of specialties	−1.17	−2.83–0.49	0.167

BMI, body mass index; zBMI/A, BMI-for-age z-score; zH/A, height-for-age z-score. Bold values indicate statistically significant results (*p* < 0.05).

### Clinical models

In the multivariable logistic regression model adjusted for age, sex, family income, physical activity level, number of medications, and number of pediatric specialties (Table 4), nutritional risk was not associated with low transition readiness (OR = 1.00, 95% CI: 0.58–1.72; *p* = 0.991). Younger age (OR = 0.82, *p* = 0.023) and male sex (female sex: OR = 0.48, *p* = 0.023) were identified as significant predictors of lower transition readiness.

**Table 4:** Multivariable logistic regression for low transition readiness.

Variable	OR	95% CI	*p*-value
Nutritional risk	1.00	0.58–1.72	0.991
Female sex	0.48	0.25–0.92	**0.023**
Age	0.82	0.70–0.96	**0.023**
Physical activity (active)	0.68	0.36–1.29	0.240
Number of medications	1.05	0.95–1.16	0.310
Number of specialties	1.08	0.92–1.26	0.340

Bold values indicate statistically significant results (*p* < 0.05).

**Figure 1:**
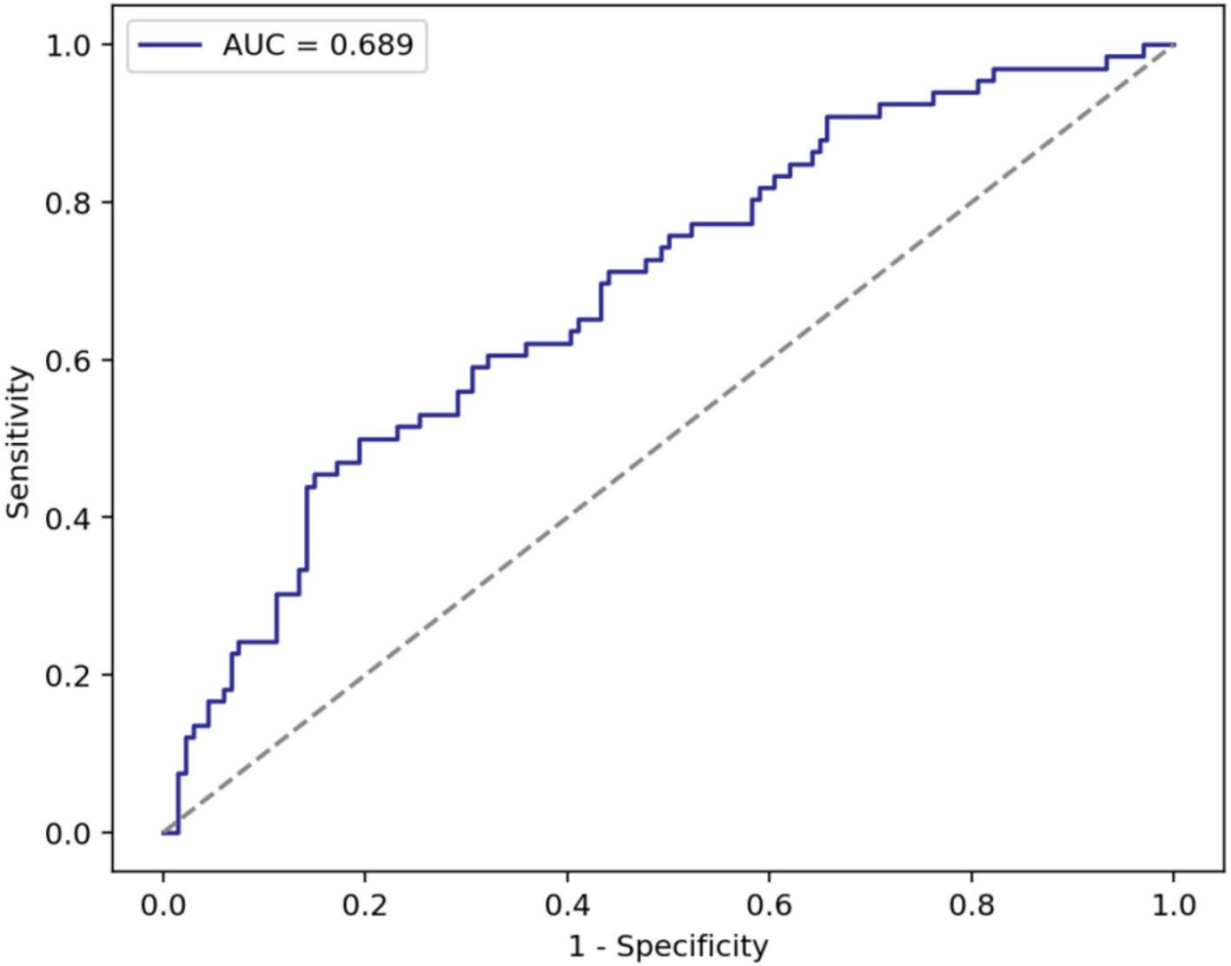
Receiver operating characteristic curve for the multivariable logistic regression model predicting low transition readiness.

The multivariable logistic regression model for low transition readiness demonstrated modest discriminatory performance, with an AUC of 0.689 (Figure 1).

### Additional analyses

**Figure 2:**
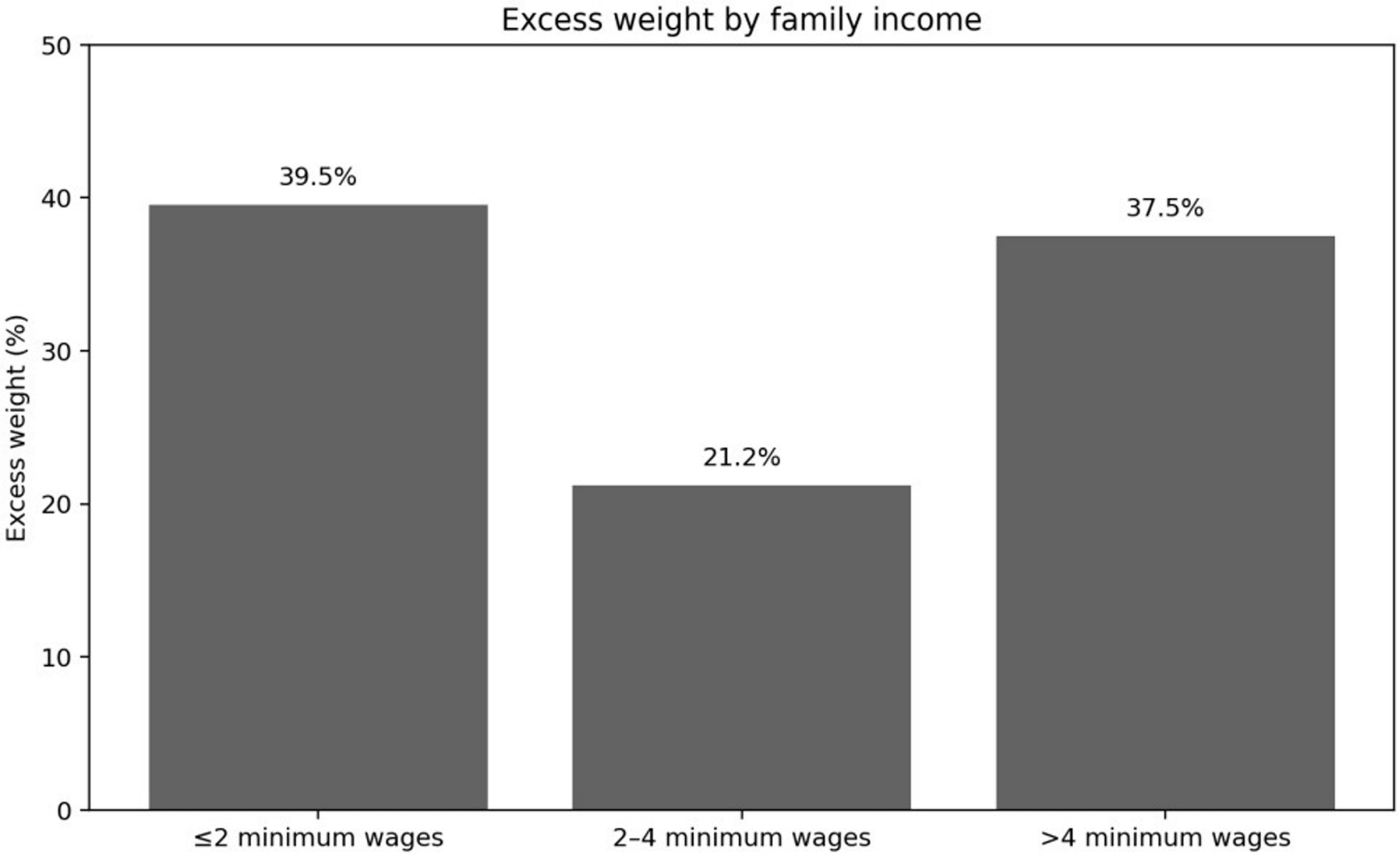
Prevalence of weight excess according to family income categories among youth with special health care needs.

Among participants with nutritional disturbances, weight excess was more prevalent in both lower- and higher-income households compared with middle-income categories (Figure 2).

## Discussion

This study evaluated the association between nutritional status and transition readiness and HRQoL in YSHCN with rare and complex chronic conditions undergoing from pediatric to adult healthcare. The main findings indicate that nutritional status, as assessed by zBMI/A, was not associated with transition readiness or HRQoL. However, linear growth (zH/A) was independently associated with better HRQoL, while female sex emerged as the strongest factor associated with both higher transition readiness and lower quality of life.

Transition readiness was primarily influenced by demographic and developmental factors rather than nutritional status. Female sex and older age were independently associated with higher TRAQ scores, reinforcing previous evidence that transition readiness reflects maturation, self-management skills, and psychosocial development rather than purely clinical or biological parameters (3, 4, 9). These findings are consistent with studies showing that readiness for transition is strongly linked to autonomy, health literacy, and engagement in care (4, 11).

The absence of an association between BMI/A and transition readiness is also conceptually plausible. The TRAQ primarily assesses behavioral competencies related to self-management, communication with healthcare providers, medication management, appointment keeping, and independent navigation of healthcare services. These competencies depend largely on cognitive maturation, psychosocial development, health literacy, and opportunities for progressive autonomy rather than on an individual’s current nutritional or metabolic status. Therefore, BMI/A may have limited influence on transition readiness.

In contrast, HRQoL demonstrated a different pattern. Although zBMI/A was not associated with HRQoL, zH/A showed a positive association, suggesting that linear growth may reflect cumulative disease burden and long-term health status. Linear growth impairment has been widely recognized as a marker of chronic illness severity in pediatric populations and has been associated with worse functional outcomes and HRQoL (12, 13, 16). This association is biologically plausible because impaired linear growth reflects the cumulative effects of chronic disease throughout childhood and adolescence. Persistent inflammation, increased metabolic demands, inadequate nutrient intake, endocrine disturbances, delayed pubertal development, and prolonged exposure to disease and its treatment may all impair linear growth over time. Consequently, lower height-for-age may represent not only previous nutritional adversity but also greater overall disease severity and cumulative physiological stress.

From a psychosocial perspective, short stature may also contribute to poorer HRQoL through reduced participation in physical and social activities, impaired self-esteem and body image, and greater dependence on caregivers, particularly during adolescence, when peer relationships and autonomy become increasingly important. These biological and psychosocial mechanisms may explain why linear growth, rather than BMI, remained independently associated with HRQoL in the present study. From a clinical perspective, these findings reinforce the importance of routine growth monitoring throughout adolescence, particularly among youth with chronic conditions undergoing healthcare transition. Assessment of linear growth may provide complementary information beyond body mass index by identifying individuals with greater cumulative disease burden who may benefit from closer multidisciplinary follow-up, including nutritional, medical, and psychosocial support.

An additional important finding was the strong and consistent association between female sex and both higher transition readiness and lower HRQoL. Although these findings may appear paradoxical, they are not necessarily contradictory. One possible explanation is that female adolescents may assume greater responsibility for self-care and health-related tasks earlier than males, potentially facilitating the development of self-management skills and transition readiness. However, this greater autonomy may also be accompanied by increased psychological demands, emotional burden, and concerns related to chronic disease management during adolescence. Consequently, greater practical readiness for transition does not necessarily translate into better perceived quality of life, particularly regarding emotional and psychosocial well-being. This interpretation is consistent with previous literature indicating that adolescent girls with chronic conditions often report worse emotional and psychosocial well-being compared to boys (17, 22–24). In addition, family income was independently associated with HRQoL, highlighting the role of social determinants of health in adolescents with chronic conditions.

Exploratory analyses suggested a higher prevalence of weight excess among participants from both lower- and higher-income households. This finding can reflect the complex nutritional transition observed in middle-income countries, where obesity increasingly coexists across different socioeconomic strata. In high-income settings, obesity is typically more prevalent among socially vulnerable populations, whereas in low- and middle-income countries, higher socioeconomic status has historically been associated with greater obesity prevalence due to increased access to ultra-processed foods and urbanized lifestyles. However, recent evidence suggests that this pattern is becoming progressively heterogeneous in developing countries (25–27).

A Brazilian study (VigiNUTRI Brasil) similarly demonstrated higher obesity prevalence among adolescents living in municipalities with higher per capita income, reinforcing the influence of contextual socioeconomic factors on adolescent nutritional outcomes. At the same time, substantial regional and social inequalities in Brazil may contribute to the coexistence of obesity among both lower- and higher-income groups, reflecting disparities in food environment, urbanization, lifestyle behaviors, and access to healthy foods (28). Although excess weight was not significantly associated with HRQoL in the present study, previous research has suggested that obesity may be associated with emotional and social challenges during adolescence, particularly in vulnerable settings and among individuals with chronic health conditions (13, 29).

Physical activity was not significantly associated with either transition readiness or HRQoL. However, the direction and magnitude of the regression coefficients remained largely unchanged after adjustment for socioeconomic factors, suggesting that potential associations were not substantially influenced by socioeconomic differences. Given its well-established benefits for physical and psychosocial health, physical activity remains an important target for health promotion among adolescents with chronic conditions (30, 31). Further studies are needed to clarify its potential role in transition readiness and HRQoL.

The predictive performance of the multivariable model was modest, with moderate discrimination and adequate calibration. These findings reinforce that transition readiness is a complex, multifactorial construct that cannot be fully explained by clinical or nutritional variables alone. Additional behavioral, psychosocial, family, and health-system factors likely contribute to transition readiness and may explain part of the variability not captured by the present model (9, 11, 32).

This study has important clinical implications. First, routine monitoring of linear growth may provide valuable information beyond BMI/A in adolescents with chronic conditions undergoing healthcare transition, as H/A appears to reflect cumulative disease burden and was independently associated with better HRQoL. Second, integrating growth monitoring with patient-reported outcomes such as HRQoL may help identify individuals who could benefit from closer multidisciplinary follow-up, including nutritional, medical, and psychosocial interventions. Finally, multidisciplinary transition programs should continue to promote comprehensive care by incorporating nutritional assessment, psychosocial support, and healthy lifestyle interventions, including physical activity, according to individual patient needs.

Some limitations should be acknowledged: the cross-sectional design precludes causal inference, and residual confounding or reverse causality cannot be ruled out. In addition, participants were consecutively recruited from multiple pediatric specialty outpatient clinics at a single tertiary referral center, which may limit the representativeness of the study population and the generalizability of the findings to other healthcare settings. Furthermore, the diagnostic heterogeneity of the sample may have influenced the findings, as YSHCN with rare and complex chronic conditions can differ considerably in disease severity, functional impairment, care complexity, and expected transition trajectories. Although the models incorporated the number of medications and pediatric specialties as proxies for clinical complexity, residual differences in disease severity and functional status were not fully captured and may have influenced the observed associations.

In addition, because a convenience sample was used and no formal sample size calculation was performed, the study may have been underpowered to detect small associations. Therefore, the absence of statistically significant associations, particularly regarding BMI-for-age and transition readiness, should be interpreted with appropriate caution.

## Conclusion

In this cohort of YSHCN with chronic conditions undergoing healthcare transition, zBMI/A was not associated with transition readiness or HRQoL. However, linear growth and family income were independently associated with better HRQoL, highlighting the importance of socioeconomic and developmental factors in patient-reported outcomes. These findings reinforce the multifactorial nature of transition readiness and HRQoL and support multidisciplinary approaches to healthcare transition. Longitudinal studies are warranted to confirm these associations and explore potential causal pathways.

## Data Availability

The raw data supporting the conclusions of this article will be made available by the authors, without undue reservation.
